# Exercise intensity and its impact on relationships between salivary immunoglobulin A, saliva flow rate and plasma cortisol concentration

**DOI:** 10.1007/s00421-018-3847-6

**Published:** 2018-04-07

**Authors:** Christof A. Leicht, Victoria L. Goosey-Tolfrey, Nicolette C. Bishop

**Affiliations:** The Peter Harrison Centre for Disability Sport, School of Sport, Exercise, and Health Sciences, Loughborough University, Loughborough, LE11 3TU UK

**Keywords:** Salivary secretory immunoglobulin A, Mucosal immune function, Exercise intensity, Exercise modality, Upper body exercise

## Abstract

**Introduction:**

Salivary secretory immunoglobulin A (sIgA), saliva flow rate and plasma cortisol concentrations have been shown to be influenced by exercise, particularly the intensity exercise is performed at, and circadian variation. The autonomic nervous system partly regulates salivary secretion, but it is not yet known whether cortisol also explains some variation in salivary parameters.

**Methods:**

Twelve moderately trained male individuals ($\dot {V}{{\text{O}}_2}$_peak legs_: 46.2 ± 6.8 mL·kg^−1^·min^−1^) performed three 45-min constant load exercise trials in the morning: arm cranking exercise at 60%$\dot {V}{{\text{O}}_2}$_peak arms_; moderate cycling at 60%$\dot {V}{{\text{O}}_2}$_peak legs_; and easy cycling at 60%$\dot {V}{{\text{O}}_2}$_peak arms_. Timed saliva samples and blood samples for plasma cortisol concentration determination were obtained before, post, 2 h post, and 4 h post-exercise. Saliva was collected in an additional resting trial at the same time points.

**Results:**

At each time point for each exercise trial, negative correlations between cortisol and saliva flow rate (explaining 25 ± 17% of the variance, *R*^2^ = 0.002–0.46) and positive correlations between cortisol and sIgA concentration (explaining 8 ± 8% of the variance *R*^2^ = 0.002–0.24) were found. Saliva flow rate increased over time, whereas sIgA concentration and cortisol decreased over time for all trials (*P* < 0.05), there was no effect of time for sIgA secretion rate (*P* = 0.16).

**Conclusion:**

These results show a relationship between cortisol and saliva flow rate, which directly impacts on the concentration of salivary analytes. This study further confirms circadian variations in salivary parameters which must be acknowledged when standardising salivary data collection.

## Introduction

Saliva flow rate and the secretion of various salivary compounds are under the control of the autonomic nervous system (Chicharro et al. 1998). Stimulation of sympathetic and parasympathetic neurons (Proctor and Carpenter 2007) or infusion of adrenergic agonists (Proctor et al. 2003) induce changes in saliva flow and the secretion of salivary secretory immunoglobulin A (sIgA). As exercise can induce changes in sympathetic and parasympathetic activity, it is a stimulus that can alter salivary secretions. For example, acute exercise of moderate intensity can increase sIgA secretion in both lower (Sari-Sarraf et al. 2007) and upper body (Leicht et al. 2011) modalities. Exercise intensity is central in altering salivary parameters, whereby exercise of demanding nature, such as interval training (Mackinnon et al. 1993) or marathon racing (Nieman et al. 2002), can reduce saliva flow rate and sIgA secretion. Salivary parameters have practical value as they are potential predictors of illness: reductions in sIgA, playing a significant part in mucosal immunity, have been associated with increased respiratory tract illness risk (Neville et al. 2008) and high training loads have been associated with a reduced sIgA secretion (Fahlman and Engels 2005; Neville et al. 2008; Leicht et al. 2012). However, the practical use is impacted by the high inter-individual variation in sIgA (Neville et al. 2008; Leicht et al. 2012), making it difficult to define universal threshold values that may be related to illness risk. Whilst dehydration (Walsh et al. 2004) or upcoming illness (Neville et al. 2010) have an impact on salivary protein concentration, to date, the source of this inter-individual sIgA variation in healthy, adequately hydrated individuals remains obscure.

Cortisol impacts directly on immune cells: For example, infusion and correlation experiments show that cortisol induces lymphopenia (Tonnesen et al. 1987) and that cortisol can reduce B cell activation and proliferation (Cupps et al. 1985). In the context of mucosal immunity, this is particularly relevant as IgA is secreted from differentiated B cells (Lamm 1998). Cortisol is also susceptible to acute exercise stress (Hayes et al. 2010): whereas low intensity exercise is generally associated with no change or even reductions in cortisol concentration (Jacks et al. 2002; Hill et al. 2008), exercise performed at intensities at or higher than ~ 60% $\dot {V}{{\text{O}}_2}$_max_ can acutely increase systemic (Hill et al. 2008) and salivary (Jacks et al. 2002; Hayes et al. 2015) cortisol concentration.

Hence, it appears that some common traits exist between cortisol and salivary parameters: they are both susceptible to exercise stress, and they play a role in host defence. However, whilst it has been reported that taxing sporting events inducing high cortisol concentrations also lead to perturbations of mucosal immunity (Nieman 1997), it is not known whether variations in cortisol concentration between individuals may be related to variations in the salivary response. The extent by which cortisol may explain some of the reported large inter-individual variations in salivary parameters (Neville et al. 2008; Leicht et al. 2012) is not known either. Furthermore, the upper body exercise modality has not received much attention in the field of mucosal immunity; however, the mucosal immune response and knowledge about the underlying mechanisms is especially important in those restricted to this modality due to their high infection susceptibility (Cardenas et al. 2004).

Therefore, this study investigates exercise of varying modality and intensity to analyse plasma cortisol concentration and salivary parameters in the subsequent 4 h period. Correlations between cortisol and salivary parameters within time points are examined. We hypothesise cortisol to explain some of the inter-individual variation in salivary parameters.

## Materials and methods

Data were obtained as part of a study published previously (Leicht et al. 2016). As a consequence, the exercise intervention, participant, and exercise characteristics are identical between those studies. The study was approved by Loughborough University’s Ethics committee. Written informed consent was obtained from all individual participants included in the study.

### Participants and experimental design

Twelve recreationally trained male individuals volunteered to participate (age: 25 ± 4 years, body mass: 76 ± 9 kg, self-reported sporting activity 4.0 ± 1.2 h·week^− 1^, $\dot {V}{{\text{O}}_2}$_peak arms_: 32.1 ± 6.0 mL·kg^−1^·min^−1^, $\dot {V}{{\text{O}}_2}$_peak legs_: 46.2 ± 6.8 mL·kg^−1^·min^−1^). The participants visited the laboratory on six occasions for two preliminary and four main trials. Initially, body mass and stature were determined using scales (model 770, seca, Birmingham, UK) and a Leicester portable stadiometer (seca, Birmingham, UK), respectively. In the two preliminary trials (visits 1 and 2), peak oxygen consumption ($\dot {V}{{\text{O}}_2}$_peak_) was determined during arm exercise (determining $\dot {V}{{\text{O}}_2}$_peak arms_) using an arm crank ergometer (Angio, Lode, Groningen, Holland) and during cycling exercise (determining $\dot {V}{{\text{O}}_2}$_peak legs_) using a cycle ergometer (Excalibur, Lode, Groningen, Holland) in a randomised order. For this, participants performed a graded exercise test to volitional exhaustion, with an initial power output of 35 W (arms) and 70 W (legs), respectively; power output was then increased every 3 min by 15 W (arms) or 30 W (legs) until exhaustion. Arm exercise was performed in a sitting position, the centre of the crank at shoulder level with arms slightly flexed at maximum reach, cycling with legs slightly flexed at maximum reach. Settings were noted and replicated for all main trials.

Main trials (visits 3–5) were performed in a randomised order after a 24 h food standardisation period, where participants consumed the same freely chosen diet for all main trials. Caffeine and exercise were not allowed 24 h before the experiments. To account for diurnal variations, exercise tests were performed in the morning (start: 07:45−09:15) for all participants and at the same time of day for each individual participant. Trials were performed at 21.9 ± 0.6 °C and at 33 ± 8% relative humidity. Main trials consisted of 45 min of steady state exercise as follows: (1) arm exercise at 60%$\dot {V}{{\text{O}}_2}$_peak arms_; (2) moderate cycling at 60%$\dot {V}{{\text{O}}_2}$_peak legs_; and (3) easy cycling at 60%$\dot {V}{{\text{O}}_2}$_peak arms_. A 5 min warm-up was performed at 50% of the start load before each condition. Oxygen uptake ($\dot {V}{{\text{O}}_2}$) was determined at 5 min intervals and power output was adjusted if necessary. For all experiments, oxygen uptake was determined over 1 min periods using Douglas bags and a gas analyser (Servomex 1440, Servomex Ltd, Crowborough, UK), and heart rate was continuously monitored using a Polar RS400 (POLAR, Kempele, Finland) monitor. Participants further indicated their rating of perceived exertion (RPE) on a scale ranging from 6 to 20 (Borg 1982). Apart from the times when gas exchange was measured water during exercise was given ad libitum. Water intake in the 4 h post-exercise passive recovery period was recorded and replicated for the remaining main trials; food and drinks other than water were not allowed during the main trials. In the recovery period, participants were allowed to perform non-strenuous activities such as reading or writing, they were generally seated but were allowed to get up to for toilet visits. A further main trial (visit 6) was performed at the same time of day (*N* = 10), which consisted of a 45-min resting period instead of the exercise intervention for the collection of saliva samples at rest.

### Salivary and plasma analyses

Saliva and plasma samples were obtained pre, post, 2 h post and 4 h post-exercise for all main trials. The pre-samples were collected > 60 min after participants had woken up. Apart from the collection immediately after exercise, participants rested on a bed for 10 min before samples were taken. Saliva samples were taken first, immediately followed by the venepuncture.

Salivary analyses were performed as explained in detail previously (Leicht et al. 2011). In brief, participants rinsed their mouth with water 10 min before each saliva collection, they then provided an unstimulated timed saliva sample with their head slightly tilted forward and minimal orofacial movement, and saliva was immediately centrifuged (2 min at 13,400 rpm). The supernatant was stored at − 20 °C for a maximum of 9 months. Saliva volume was estimated assuming saliva density to be 1.00 g·mL^− 1^ (Cole and Eastoe 1988), and saliva flow rate was calculated from saliva volume and collection time. The sIgA concentration was determined in duplicate by sandwich enzyme-linked immunosorbent assay (ELISA), the within assay coefficient of variation being 2.8 ± 3.5%.

For venous blood sample collection, participants were lying in a supine position. Blood was collected into K_3_EDTA monovette containers (Sarstedt, Nuembrecht, Germany) from a superficial vein of the forearm, drawn by separate venepunctures for each sample. Haematocrit was immediately determined for each sample using an automated haematology analyser (Coulter Ac·T 5diff OV; Beckman Coulter, High Wycombe, UK). Following centrifugation (10 min at 3000 rpm and 4 °C), plasma was stored at − 20 °C until analysis. Cortisol concentration was determined in duplicate by ELISA according to the manufacturer’s instructions (DRG Instruments GmbH, Marburg, Germany), with a within assay coefficient of variation of 2.7 ± 2.7%.

### Statistical analyses

The SPSS 23.0 statistical package (SPSS Inc., Chicago IL, USA) was used for all statistical analyses. Means and standard deviations were computed for all variables, and normality was checked with the Shapiro Wilk test. Non-normal data were converted using square root (saliva flow rate) or logarithmic (cortisol, sIgA concentration and sIgA secretion rate) transformations which achieved normality for all variables. A repeated measures two-way (trial, time) analysis of variance (ANOVA) was conducted on cortisol, saliva flow rate, sIgA concentration and sIgA secretion rate. Huynh-Feldt corrections were applied when sphericity was violated and Sidak adjustments applied for post hoc comparisons. Pearson’s correlation coefficients (*R*) and coefficients of determination (*R*^2^) were determined for bivariate relationships (cortisol vs saliva flow rate; cortisol vs sIgA concentration), and uncorrected *P* values for these relationships are reported. Physiological exercise descriptors were analysed using a one-way (trial) repeated measures ANOVA or the non-parametric equivalents for non-normal and RPE data. Statistical significance was accepted at *P* < 0.05.

## Results

### Salivary parameters and plasma cortisol: relationships

**Fig. 1:**
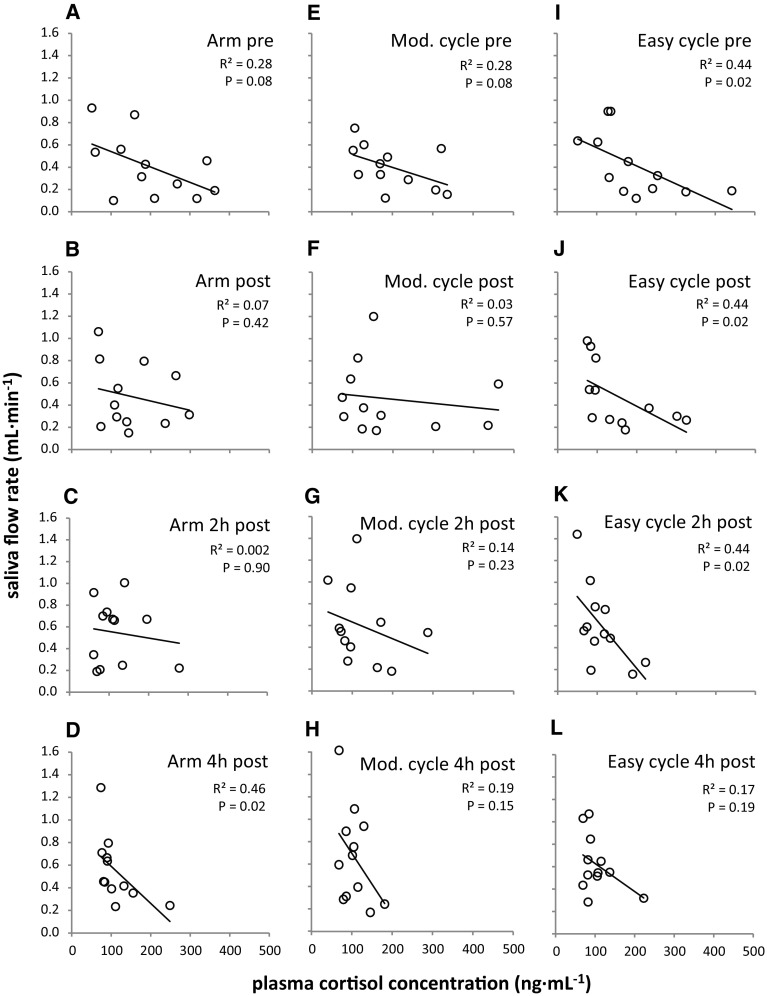
Correlations analysis of plasma cortisol concentration vs saliva flow rate for arm exercise, moderate (Mod.) and easy cycling pre, post, 2 h post and 4 h post-exercise. Raw data and linear regression lines

Negative relationships between plasma cortisol concentration and saliva flow rate were found for all trials at all time points (Fig. 1). Most common variation between the two variables across time points was observed during the easy cycle trial (Fig. 1I–K, *P* < 0.05), whereas the common variation post-exercise and 2 h post-exercise was markedly reduced for arm exercise (Fig. 1B, C) and the moderate cycle trial (Fig. 1F, G).

Correlation coefficients between plasma cortisol concentration and sIgA concentration were predominantly positive but were considerably lower (*R*^2^ up to 0.24; Table 1) than those found for the cortisol – saliva flow rate relationships (*R*^2^ up to 0.46; Fig. 1); none of these correlations reached significance (*P* > 0.05).

**Table 1:** Bivariate correlations of plasma cortisol concentration vs sIgA concentration

Time point	Trial		
	Arm exercise	Moderate cycle	Easy cycle
Pre	*R* = 0.34, *R*^2^ = 0.12	*R* = − 0.04, *R*^2^ = 0.002	*R* = 0.07, *R*^2^ = 0.004
	*P* = 0.28	*P* = 0.90	*P* = 0.84
Post	*R* = 0.44, *R*^2^ = 0.19	*R* = 0.24, *R*^2^ = 0.06	*R* = 0.20, *R*^2^ = 0.04
	*P* = 0.15	*P* = 0.46	*P* = 0.53
2 h Post	*R* = 0.24, *R*^2^ = 0.06	*R* = 0.11, *R*^2^ = 0.01	*R* = 0.15, *R*^2^ = 0.02
	*P* = 0.46	*P* = 0.73	*P* = 0.64
4 h Post	*R* = 0.49, *R*^2^ = 0.24	*R* = 0.25, *R*^2^ = 0.06	*R* = 0.32, *R*^2^ = 0.10
	*P* = 0.11	*P* = 0.44	*P* = 0.31

### Salivary parameters and plasma cortisol: time and trial effects

**Fig. 2:**
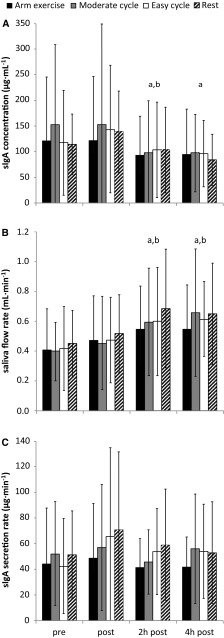
Salivary responses (mean ± SD). Significant difference between ^a^pre and ^b^post (*P* < 0.05)

**Fig. 3:**
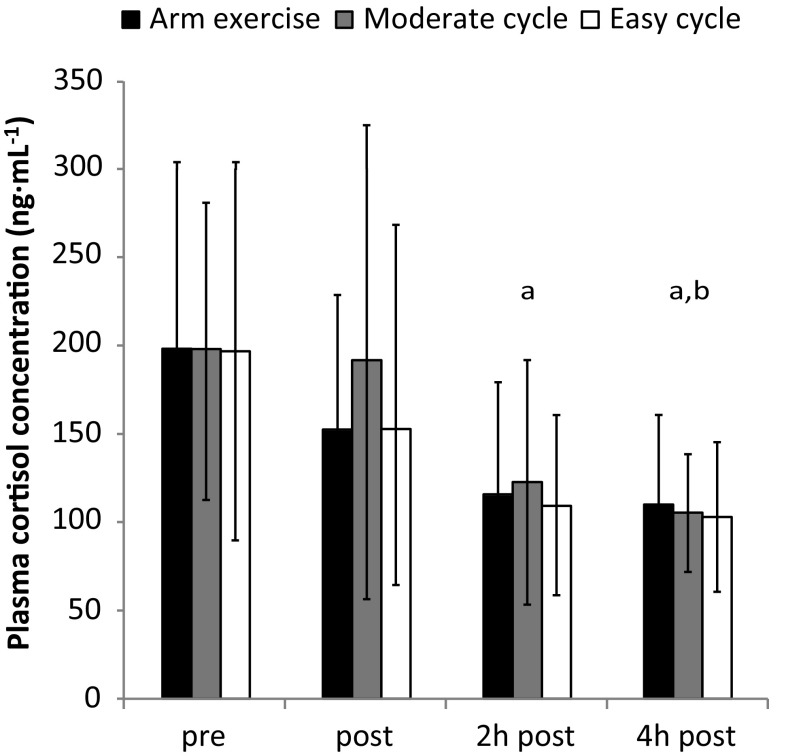
Plasma cortisol responses (mean ± SD). Significant difference between ^a^pre and ^b^post (*P* < 0.05)

SIgA concentration was decreased in the recovery period (main effect of time: *P* < 0.001, Fig. 2A), whilst saliva flow rate increased as time progressed (main effect of time: *P* < 0.001, Fig. 2B) with higher values in the recovery period. For all trials, sIgA secretion rate did not change over the course of the study (main effect of time: *P* = 0.16; Fig. 2C). No effect of trial was found in any of the salivary parameters (*P* = 0.47–0.83), indicating that exercise did not alter these parameters when compared to the resting trial. The plasma cortisol concentration did not differ between trials (*P* = 0.30) and decreased over time, with a significant reduction at 2 and 4 h post (*P* < 0.001; Fig. 3).

### Exercise response

The exercise trials resulted in distinctively different physiological and psychophysiological responses, the lowest power output, heart rate and RPE values found for easy cycling. Arm exercise and easy cycling did not differ with regards to absolute oxygen uptake; arm exercise and moderate cycling did not differ with regards to relative oxygen uptake (*P* > 0.05; Table 2). Haematocrit did not differ significantly between trials (*P* = 0.23; Table 3).

**Table 2:** Physiological and psychophysiological exercise descriptors

Parameter	Trial		
	Arm exercise	Moderate cycle	Easy cycle
Power output (W)	73 ± 14*	148 ± 30*	101 ± 25*
Heart rate (*b*·min^−1^)	141 ± 9*	150 ± 12*	123 ± 11*
Average RPE	13.3 (12.6, 13.9)	13.2 (12.7, 14.0)	10.6 (10.0, 11.0)*
$\dot {V}{{\text{O}}_2}$ (L·min^−1^)	1.50 ± 0.28	2.16 ± 0.34*	1.50 ± 0.28
%$\dot {V}{{\text{O}}_2}$_peak arms_	62.3 ± 1.4	–	62.3 ± 1.1
%$\dot {V}{{\text{O}}_2}$_peak cycling_	–	62.3 ± 1.0	43.2 ± 5.4*

Data indicate mean ± SD or median (lower quartile, upper quartile)

*RPE* rating of perceived exertion

*Significant difference to both other trials (*P* < 0.05)

**Table 3:** Haematocrit (%) in response to the four main trials

Time point	Trial			
	Arm exercise	Moderate cycle	Easy cycle	Rest
Pre	43.0 ± 2.7	43.3 ± 2.7	43.1 ± 3.0	41.1 ± 2.9
Post	45.0 ± 2.9*	44.9 ± 3.0*	44.3 ± 2.7*	43.1 ± 2.3*
2 h Post	43.3 ± 2.9	43.7 ± 3.1	43.5 ± 3.3	41.9 ± 2.3
4 h Post	42.9 ± 3.1	43.0 ± 2.4	43.1 ± 2.7	42.1 ± 2.5

*Significant difference to other time points (*P* < 0.05)

## Discussion

### Main findings

This is the first study to analyse salivary parameters and plasma cortisol concentration and their relationship for different exercise intensities/modalities for an extended post-exercise recovery period of 4 h. The main finding was that plasma cortisol concentration explained some of the variance found in saliva flow (Fig. 1). This adds to the body of knowledge that has so far mainly focussed on neural regulation of salivary secretions (Chicharro et al. 1998; Proctor and Carpenter 2007). Second, increases in saliva flow rate were found over time, but sIgA secretion rate remained unchanged as this was accompanied by decreases in sIgA concentration (Fig. 2). These responses were also found in the resting trial and were not affected by exercise, a confirmation of earlier work indicating the presence of circadian rhythms for salivary markers (Dawes 1972, 1975). Finally, sIgA concentration decreases were accompanied by cortisol concentration decreases over time, and the relationships between those markers were mainly positive (Table 1). However, none of these relationships reached significance, implying that cortisol is not an appropriate surrogate marker for sIgA concentration and vice versa.

### Cortisol and its relationship with saliva flow

The present findings show that some of the inter-individual variation of saliva flow rate can be explained by plasma cortisol concentration. This adds to earlier research comparing plasma cortisol and salivary parameters, showing the parallel fall in sIgA concentration and cortisol in the hours after awakening (Hucklebridge et al. 1998). Importantly, the present findings do not only support the established facts that circadian variations exist for cortisol (Dimitrov et al. 2009), saliva flow rate (Dawes 1975) and sIgA concentration (Li and Gleeson 2004), with the fall of cortisol coinciding with the rise in saliva flow rate and the fall in sIgA concentration during the course of the day. It is crucial to note that a relationship between cortisol and saliva flow rate can be observed between participants for individual time points as well (hence excluding the factor circadian variation). This means that individuals with high cortisol concentrations tend to exhibit lower saliva flow rates. It is furthermore interesting that the negative relationships between cortisol concentration and saliva flow rate are less pronounced immediately post and 2 h post the two trials performed at moderate exercise intensity (arm exercise: Fig. 1B, C and moderate cycling: Fig. 1F, G). This is in contrast to easy cycling, where clear negative relationships are found throughout the analysed time points (Fig. 1I–L). Even though not measured in the current study, we speculate that the exercise intensity of these two trials was high enough to stimulate factors known to modulate saliva flow, such as adrenergic or cholinergic pathways (Proctor et al. 2003) or increased autonomic nerve activity (Carpenter et al. 2000), hence overriding the modulating effect of cortisol alone. The present correlations analysis also shows that easy cycling serves as an appropriate control trial with respect to the cortisol/saliva flow relationships, as they appear to remain unaffected by this exercise intervention.

The implication of cortisol in the regulation of saliva flow seems intriguing and the presented data provide a good starting point to discuss this suggestion. As many salivary parameters are expressed as secretion rates which take saliva flow rate into account, further investigations into the potential modulating role of cortisol have direct relevance to the investigator of salivary secretions. It is worth pointing out that cortisol has not been considered to be potentially involved in the regulation of saliva flow previously; which has so far been attributed to autonomic neural regulation only (Chicharro et al. 1998; Proctor and Carpenter 2007). Nonetheless, the evidence presented here is correlational in nature, and causal evidence must now be obtained to substantiate this suggestion. This may involve cortisol infusion experiments to answer the cause and effect question with greater certainty and to establish the extent by which cortisol may modulate salivary secretions.

Approaching the problem from an alternative, exercise-based angle, future research may consider focussing on conducting exercise interventions in the afternoon. At this time, cortisol concentrations are relatively stable (Hucklebridge et al. 1998; Dimitrov et al. 2009) and do not fall following their circadian rhythm as they do in the morning, which may cancel out the effects of an intervention aimed at increasing cortisol concentrations. Exercise in the afternoon may hence induce a more pronounced rise in cortisol concentrations (Dimitriou et al. 2002), allowing the investigation of its effect on saliva flow and salivary secretions. It has also been suggested that the diurnal variation of cortisol and testosterone may impact on strength training adaptations: training performed in the afternoon is possibly more effective due to lower cortisol concentrations and more pronounced testosterone responses at this time of day (Hayes et al. 2010). Similarly, it could be speculated that performing exercise in the afternoon with its greater capacity to increase cortisol levels has a more pronounced impact on immunity than exercise in the morning. However, this must be substantiated with future experiments, as in the case of mucosal immunity, the limited existing evidence does not support this speculation (Dimitriou et al. 2002).

### Moderate intensity exercise and salivary secretory immunoglobulin A

Previous data suggest that submaximal exercise can increase sIgA secretion rate (Sari-Sarraf et al. 2007; Leicht et al. 2011). A better protection against invading pathogens could hence be hypothesised based on the finding that reductions in sIgA increase the upper respiratory infection risk (Neville et al. 2008). However, these aforementioned studies (Sari-Sarraf et al. 2007; Leicht et al. 2011) were limited to a post-exercise monitoring period of up to 30 min. This short post-exercise observation period may have been a limitation that the current study aimed to overcome, and one of the aims of the present study was hence to monitor salivary parameters over a relatively long post-exercise period to establish how long any effects of acute submaximal exercise may last.

None of the exercise interventions in the current study significantly altered any of the salivary parameters in comparison to the responses observed in the resting trial. In light of this, it is important to point out that the literature is ambiguous with respect to the acute sIgA secretion response to exercise, with sIgA secretion rate reported to increase (Sari-Sarraf et al. 2007; Leicht et al. 2011) or to remain unaffected (Bishop et al. 2000; Reid et al. 2001; Sari-Sarraf et al. 2006; Davison 2011) by submaximal exercise interventions. Interestingly, sIgA concentrations decreased over time, with higher values in the morning, falling over the course of the day. These reductions in sIgA concentration over time can be related to the increase in saliva flow causing a diluting effect. Saliva flow rate hence affects sIgA concentration without affecting sIgA secretion rate, which is in line with existing literature (Bishop et al. 2000; Reid et al. 2001). Whilst the present results do not confirm an increase in sIgA secretion rate for any exercise trial, they confirm previous work (Davison 2011) pointing out the importance to consider circadian variations in markers of mucosal immunity. Previous evidence investigating sIgA metabolism (MacKinnon and Jenkins 1993; Reid et al. 2001; Sari-Sarraf et al. 2006) without accounting for circadian variation may hence have to be revisited, especially when investigating exercise of a long duration (Bishop et al. 2000; Nieman et al. 2002) where such variation may have the largest impact.

A final aim of the current study was to investigate the effect of exercise modality. This is an important issue should previous findings around mucosal immunity that were predominantly derived from lower body exercise modalities (Bishop et al. 2000; Reid et al. 2001; Sari-Sarraf et al. 2006, 2007; Davison 2011) be applied to individuals restricted to upper body exercise modalities. It is worth noting that whilst some studies have investigated acute mucosal immune responses for upper body exercise modalities (Leicht et al. 2011; Allgrove et al. 2012), we are not aware of studies comparing upper and lower body exercise modalities. With the caveat that exercise did not significantly alter markers of mucosal immunity in the present study, it is worth pointing out that there was also no difference in any salivary markers *between* exercise modalities (Fig. 2). This provides a good base for future investigations comparing these modalities, which should now study interventions that do induce changes in mucosal immunity affecting host protection, for example, by altering sIgA metabolism. Even though the current protocol was designed to fulfil this purpose, we must again stress that sIgA secretion rate was unaffected by exercise. For future investigations, we suggest to increase the exercise duration and/or intensity to reach this aim.

### Limitations

This study employed an a posteriori design for data analysis (i.e., using data from a previously published study). Further confirmation of the present findings should be obtained using a priori study designs. To account for circadian variation more rigorously, future studies may want to narrow the testing start time to a smaller window than the one presently chosen (90 min). Future studies are also encouraged to record and document data such as waking time, and relate data collection time points to waking time, especially relevant when tracking cortisol or sIgA responses immediately after awakening (Hucklebridge et al. 1998). We did not record these data in detail as all first samples were collected > 30 min following awakening, which is the time frame after which the acute awakening response of sIgA and cortisol appears to level off (Hucklebridge et al. 1998).

## Conclusion

This study shows that plasma cortisol concentration explains some of the inter-individual variance found in saliva flow. Furthermore, the results demonstrate that circadian effects must be considered for salivary flow rate, which impacts on the concentration of any salivary compounds such as sIgA.

## Abbreviations

ANOVA: Analysis of variance
ELISA: Enzyme-linked immunosorbent assay
*R*: Correlation coefficient
*R*^2^: Coefficient of determination
RPE: Rating of perceived exertion
SD: Standard deviation
sIgA: Salivary secretory immunoglobulin A
$\dot {V}{{\text{O}}_2}$_max_: Maximum oxygen uptake
$\dot {V}{{\text{O}}_2}$_peak_: Peak oxygen uptake
